# Evidence for moiré intralayer excitons in twisted WSe_2_/WSe_2_ homobilayer superlattices

**DOI:** 10.1038/s41377-022-00854-0

**Published:** 2022-06-01

**Authors:** Biao Wu, Haihong Zheng, Shaofei Li, Junnan Ding, Jun He, Yujia Zeng, Keqiu Chen, Zongwen Liu, Shula Chen, Anlian Pan, Yanping Liu

**Affiliations:** 1 School of Physics and Electronics, Hunan Key Laboratory for Super-microstructure and Ultrafast Process, Central South University, 932 South Lushan Road, Changsha, Hunan 410083 China; 2 State Key Laboratory of High-Performance Complex Manufacturing, Central South University, 932 South Lushan Road, Changsha, Hunan 410083 China; 3 Department of Applied Physics, School of Physics and Electronics, Hunan University, Changsha, 410082 China; 4 School of Chemical and Biomolecular Engineering, The University of Sydney, Sydney, NSW 2006 Australia; 5 The University of Sydney Nano Institute, The University of Sydney, Sydney, NSW 2006 Australia; 6 Hunan Institute of Optoelectronic Integration, College of Materials Science and Engineering, Hunan University, Changsha, Hunan 410082 China; 7 Shenzhen Research Institute of Central South University, A510a, Virtual University Building, Southern District, High-tech Industrial Park, Yuehai Street, Nanshan District, Shenzhen, China

**Keywords:** Photonic crystals, Photonic devices

## Abstract

Recent advances in twisted van der Waals heterostructure superlattices have emerged as a powerful and attractive platform for exploring novel condensed matter physics due to the interplay between the moiré potential and Coulomb interactions. The moiré superlattices act as a periodic confinement potential in space to capture interlayer excitons (IXs), resulting in moiré exciton arrays, which provide opportunities for quantum emitters and many-body physics. The observation of moiré IXs in twisted transition-metal dichalcogenide (TMD) heterostructures has recently been widely reported. However, the capture and study of the moiré intralayer excitons based on TMD twisted homobilayer (T-HB) remain elusive. Here, we report the observation of moiré intralayer excitons in a WSe_2_/WSe_2_ T-HB with a small twist angle by measuring PL spectrum. The multiple split peaks with an energy range of 1.55–1.73 eV are different from that of the monolayer WSe_2_ exciton peaks. The split peaks were caused by the trapping of intralayer excitons via the moiré potential. The confinement effect of the moiré potential on the moiré intralayer excitons was further demonstrated by the changing of temperature, laser power, and valley polarization. Our findings provide a new avenue for exploring new correlated quantum phenomena and their applications.

## Abstract (web summary)

The moiré intralayer excitons in the twisted WSe_2_/WSe_2_ homobilayers superlattices were successfully observed and confirmed the existence of moiré intralayer excitons.

## Graphical abstract

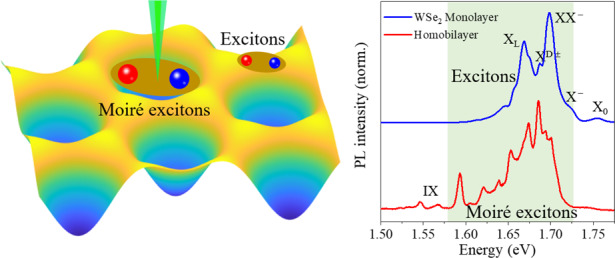

## Introduction

Two-dimensional (2D) moiré superlattices are opening up new opportunities for exploring novel correlated physics fundamentals, since almost flat electron bands can be designed to enhance the influence of electron-electron correlation^1–5^. This phenomenon was observed for the first time in graphene-based moiré superlattices, where the graphene moiré systems displayed correlated insulator states, topological phases and unconventional superconductivity^6–8^. Promoted by the graphene-based moiré superlattices, it was predicted that the moiré superlattice based on TMD may have flatter minibands that improve the effect of long-range Coulomb interactions^9–11^. In contrast to the twisted bilayer graphene, the flat bands only appear at magic angles^12^, while the twisted TMD bilayer has a wider range of angles^10,13^. Therefore, moiré superlattices based on twisted TMD materials can provide a new dimension to explore strongly correlated novel quantum phenomena, such as the quantum emitters and correlated insulators^14–16^.

Moiré superlattices are formed by vertically stacking two monolayers of 2D materials with a small twist angle or lattice mismatch^17^. The moiré potential acts as an ordered array of nanodots in real space by modulating the electronic structure, which essentially modifies the properties of the IXs^18,19^. In addition, the electronic band structure of the moiré superlattice can be designed by the stacking angle, thereby modulating the moiré period and the interaction between the moiré excitons^20,21^. Therefore, moiré superlattices based on TMD offer a powerful platform for quantum electronics and optics^4,5^. Recently, the features of IX trapped in moiré potential have been extensively reported in TMD heterostructure samples^17–20^. The moiré potential acts as a periodical trap potential in space to confine the IXs, resulting in moiré exciton arrays^20^. However, reports on moiré intralayer excitons in the TMD T-HB have been absent thus far.

In this work, we report the successful preparation of a WSe_2_/WSe_2_ T-HB with an interlayer twist angle approaching zero by the tear-and-stack technique^7,22^. We measured and fitted the second-harmonic generation (SHG) signals of both WSe_2_ monolayers at the top and bottom of the sample, and determined that the twist angle between the WSe_2_ bilayers was 1.36 ± 0.05. The PL spectrum at 8 K in the T-HB region was fitted by the Lorentzian function to obtain 8 small split peaks that are different from exciton peaks of the monolayer WSe_2_. The energy range of the split peaks was 1.55–1.73 eV, which was attributed to the flat minibands produced by the moiré potential modulation on the electronic structure. In addition, we were able to control the moiré intralayer excitons through the variation of temperature, laser power, and valley polarization, thus achieving the confining effect of the moiré potential on the moiré intralayer excitons. Our results provide a promising opportunity for further exploring new condensed matter physics and its applications.

## Results

**Fig. 1:**
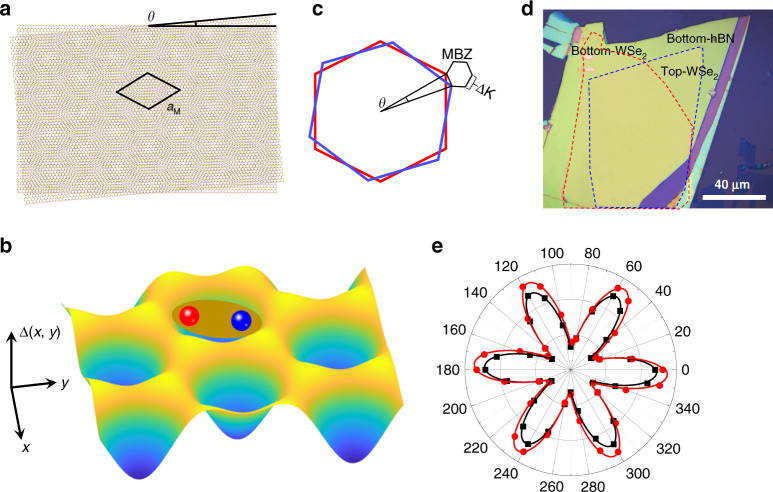
Optical properties of moiré superlattice in twisted homobilayer. **a** Schematic depiction of the moiré superlattice structure generated by the WSe_2_ bilayer with a twist angle of *θ*, where *a*_M_ is the moiré potential period. **b** The moiré superlattice results in periodic modulation of the electrostatic potential in the twisted homobilayer, which can trap excitons in the periodic potential. **c** The formation of the moiré unit cell in the twisted homobilayer causes the formation of the moiré Brillouin zone. **d** Optical image of the WSe_2_/WSe_2_ twisted homobilayer placed on hexagonal boron nitride (hBN). The bottom WSe_2_ and top WSe_2_ monolayer regions are represented by red and blue dashed lines, respectively. **e** Measured and fitted the SHG signals of the WSe_2_ monolayer area on the top and bottom of the sample to determine that the twist angle between the WSe_2_ bilayers is close to zero

The schematic diagram of the moiré superlattice formed by the WSe_2_/WSe_2_ T-HB with a small twist angle *θ* is shown in Fig. 1a. The period of the moiré superlattice is *a*_M_ = *a*/(2 sin[*θ*/2]), where *a* is the lattice constant of the WSe_2_ monolayer. Figure 1b presents a schematic of the spatial periodic moiré potential generated by the moiré superlattice in Fig. 1a. The in-plane momentum of the system is defined as the high energy level of the moiré potential. The center-of-mass motion of excitons in the moiré potential can be expressed by an effective Hamiltonian^19^

1 $$H = - \hbar \Omega _0 + \frac{{\hbar ^2k^2}}{{2M}} + \Delta (r)$$

where ℏΩ_0_ is the energy constant of the lowest exciton state energy, *M* represents the effective mass of exciton, *ħ*^2^*k*^2^/(2*M*) is the kinetic energy, and Δ(*r*) is the moiré potential energy. The spatial periodic potential, Δ(*r*) = Δ(*x*, *y*), can modulate the undisturbed electron band of the monolayer, forming many flat electron minibands, thereby changing the energy band structure of the twisted bilayer. As a consequence, depending on the moiré potential, electrons or holes, and light-excited quasi-particles, such as excitons and exciton complexes may be trapped in the potential. Figure 1c shows the mini-Brillouin zone formed in the WSe_2_ bilayer with a rotation angle of *θ*. The size of the Brillouin zone determines the number of moiré cells occupied and the intensity of the optical transition dipole. Figure 1d illustrates the optical image of the sample with the WSe_2_/WSe_2_ T-HB, in which the red and blue dashed areas represent the bottom and top monolayers, respectively. The samples were fabricated following a mechanical exfoliation and tear-and-stack technique. The polarization-dependent SHG signals were measured to determine the twist angle between layers. As shown in Fig. 1e, the fitted data indicate that the interlayer twist angle of the T-HB is close to zero (1.36 ± 0.05°).

**Fig. 2:**
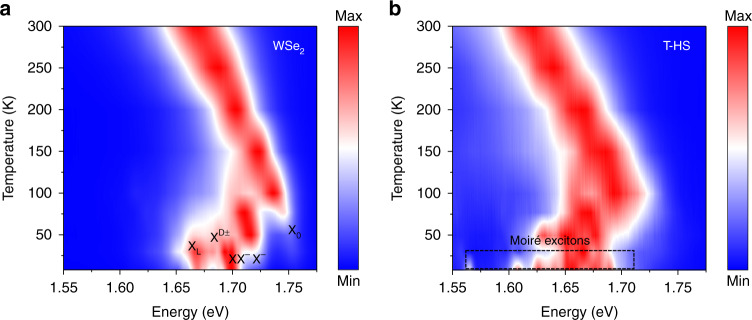
Temperature-dependent PL spectra of WSe_2_/WSe_2_ twisted homobilayer. **a** The normalized PL spectra of monolayer WSe_2_ versus temperature (8 K~300 K). As the temperature decreases, the PL spectrum shows a blue shift, and the types of excitons become more complicated. The energies from high to low are neutral exciton, charged exciton, charged biexciton, dark exciton, and local exciton. **b** The normalized PL spectrum of T-HB varies with temperature, from 8 K to 300 K. At 8 K, the PL spectrum of the T-HB region showed many small split peaks different from the exciton peaks of the monolayer WSe_2_ exciton peaks, indicating that the moiré exciton peaks formed by the trapping of intralayer excitons are affected by the moiré potential

To better understand the PL characteristics of the T-HB, we studied the normalized PL spectra of the monolayer and the T-HB region as a function of temperature. Figure 2a shows the PL spectrum of the WSe_2_ monolayer with temperature, varying from 8 to 300 K. At 300 K, there is only one peak in the PL spectrum of the WSe_2_ monolayer, and its energy is 1.669 eV. As the temperature decreases, the emission peak exhibits a blue shift, which is consistent with the Varshni equation describing the change of the bandgap of various semiconductors with temperature^23^. In addition, it can be clearly observed that the spectral intensity of the monolayer WSe_2_ shifts neutral exciton (X_0_) to charged exciton (X^−^), and then to charged biexcitons (XX^−^), which is caused by the reduction of electron thermal fluctuations^24,25^. At 8 k, there are abundant excitons in the PL spectrum, and the energies from high to low are X_0_, X^−^, XX^−^, dark exciton (X^D±^) and local exciton (X_L_), which can be identified by PL spectrum^26,27^. Figure 2b presents the results of the temperature-dependent PL for the T-HB. As the temperature cools, the emission peak appears blue shift. Noticeably, when the temperature is 8 K, the PL spectra show many small splitting peaks different from the WSe_2_ monolayer exciton peaks, which is caused by the moiré potential in the T-HB.

**Fig. 3:**
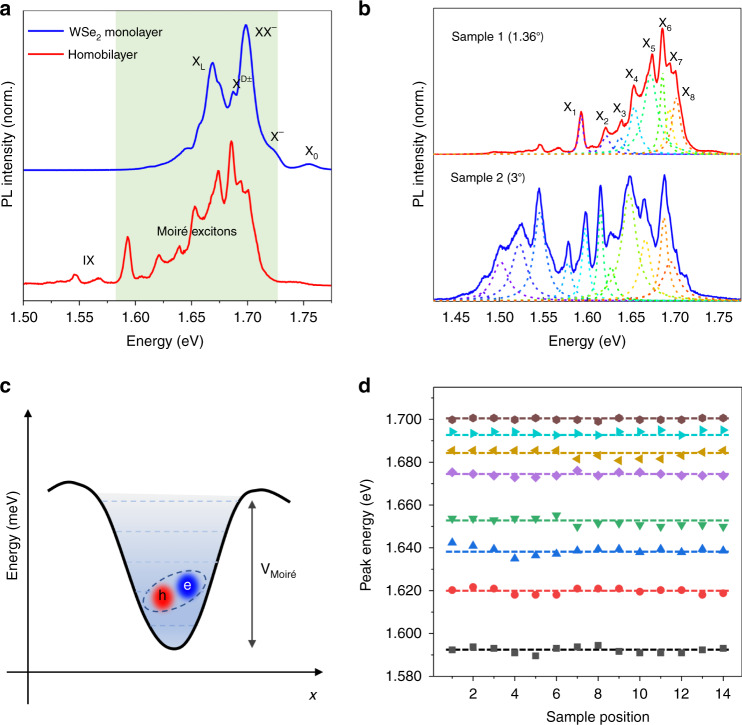
Optical characteristics of moiré intralayer excitons in WSe_2_/WSe_2_ twisted homobilayer. **a** Comparing the PL spectra of WSe_2_ monolayer and homobilayer region at 8 K, there are many small split peaks in the twisted bilayer, the energy position of which is different from that of the monolayer, and interlayer excitons appear in the low energy region. **b** PL spectra of homobilayers with twist angles of 1.36° and 3°. Many split small peaks are fitted by Lorentzian function, and the dotted lines of different colors represent different peaks. **c** Illustration of the spatial variation of the moiré potential (V_Moiré_) and the trapped intralayer excitons. **d** The central energy values of the splitting small peaks were extracted from the PL spectra at different positions of the homobilayer with a twist of 1.36°

To further explore the optical properties of the T-HB, we compared the PL spectra of the monolayer and the T-HB region at 8 K as shown in Fig. 3a. It can be clearly observed that the PL spectrum of the T-HB region shows two distinct IX peaks in the low energy range. The appearance of IX peaks indicates that the two monolayers of WSe_2_ were coupled very well. Interestingly, the multiple split peaks at 1.55–1.73 eV are different from the monolayer WSe_2_ exciton peaks, indicating the presence of moiré superlattices in our sample, which is caused by the trapping of intralayer excitons by the moiré potential^19,28^. For the WSe_2_/WSe_2_ T-HB with a small twist angle, the optical transition of excitons is significantly different from the WSe_2_ monolayer, due to the formation of the moiré Brillouin zone that causes the formation of flat minibands in T-HB. Figure 3b shows the PL spectra of homobilayers with twist angles of 1.36° and 3°, with 8 and 12 small peaks obtained by Lorentzian function fitting, respectively. For the WSe_2_/WSe_2_ homobilayer with a twist angle of 1.36°, the central emission energies extracted from the fitted eight small splitting peaks for the homobilayer with a twist angle of 1.36° are 1.593, 1.621, 1.638, 1.653, 1.672, 1.686, 1.694, and 1.702 eV. The moiré potential depths of the 1.36° and 3° twisted homobilayers in our experiments are 109 and 215 meV, respectively, which are similar to the results previously reported by Tran et al.^19^. As the twist angle increases, the moiré superlattice period decreases. The moiré potential is inversely proportional to the moiré superlattice period, so the moiré potential increases with the twist angle. The experimental results show that the homobilayer with a larger twist angle has more splitting and broader energy peaks, which is consistent with the theoretical results^29^. Figure 3c depicts a schematic diagram of the spatial variation of the moiré potential and the trapping of intralayer excitons. The formation of the moiré Brillouin zone in Fig. 1c changes the electronic structure in the T-HB, resulting in many flat minibands, which are marked in the moiré potential with dashed lines. In WSe_2_/WSe_2_ T-HB with a small twist angle, the Bohr radius of excitons is larger than the lattice constant of monolayer WSe_2_, but smaller than the moiré period^19^. Therefore, the intralayer excitons can be regarded as particles moving slowly in the moiré potential, which appear as small split peaks on the PL spectrum. Figure 3d shows the central energies of eight small split peaks extracted from the PL spectra at different positions in the T-HB region, indicating that the PL spectra can be repeated at different positions in the T-HB.

**Fig. 4:**
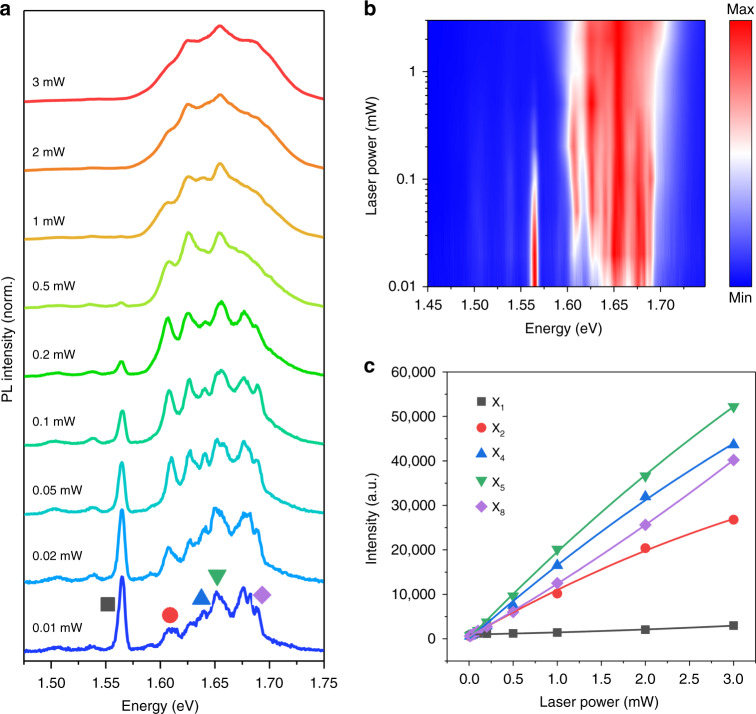
Power-dependent PL properties of WSe_2_/WSe_2_ twisted homobilayer. **a** The normalized PL spectra of different excitation powers range from 0.01 to 3 mW, the excitation photon energy is 2.33 eV, and the energy resolution is 3 meV. **b** The normalized PL spectrum of T-HB as a function of power (0.01 mW~3 mW), and the data come from Fig. 4a. **c** The geometric shapes of different colors correspond to the positions of different energy peaks, from Fig. 4a. The PL intensity of different peak positions under different excitation powers

To obtain more detailed information about the moiré potential, we investigated the power-dependent PL spectrum in the T-HB region at 8 K. Figure 4a depicts the PL spectra at different excitation powers from 0.01 to 3 mW at an excitation wavelength of 532 nm. Under the low excitation power (<0.2 mW), the shape of the PL spectrum remains basically unchanged, and each small split emission peak in PL spectrum can be clearly observed. With the increase of the power, the flat minibands in the moiré potential are gradually filled by trapping excitons and finally reach saturation, which appears as a decrease in exciton peaks and an increase in peak width on the PL spectrum. The results show that as the power increases, the modulation of the intralayer excitons is weakened by the moiré potential^20^. Figure 4b presents the normalized PL spectrum as a function of power, from 0.01 to 3 mW. The color plot, using data from Fig. 4a, makes it more intuitive to observe the evolution of excitons with power. It can be clearly seen that as the power increases, the X_1_ energy level is gradually filled and finally reaches saturation, and the moiré excitons change from multiple small split peaks to the main peak dominated by intralayer excitons. The results show that there are many small split peaks in the PL spectrum at low power, indicating that there is a confinement potential for trapping intralayer excitons, caused by the flat minibands in the moiré Brillouin zone. Figure 4c quantitatively studied the PL intensity of some peaks extracted from the PL spectrum as a function of power. The shapes of different colors indicate the different peak positions which are derived from Fig. 4a. It can be observed that the X_1_ exciton level at low power is quickly filled to reach saturation (>0.1 mW). Notably, when the power is >1 mW, the PL intensity of the X_1_ peak appears to be enhanced, which is due to the influence of the PL spectra of interlayer excitons and moiré excitons with similar energies. As the power increases, the flat minibands of higher energy levels are sequentially filled and gradually reach saturation. Exciton filling is sequentially from low energy level to high energy level. While X_8_ is at the highest energy level, and there is no sign of saturation at 3 mW, so the X_8_ peak continues to grow rapidly with the increase of power, which is consistent with previous report^20^. The results show that the relaxation and transfer of moiré excitons from higher energy levels to lower energy levels are effective at low power, and the moiré potential gradually loses its modulation effect of intralayer excitons as the power increases.

## Discussion

**Fig. 5:**
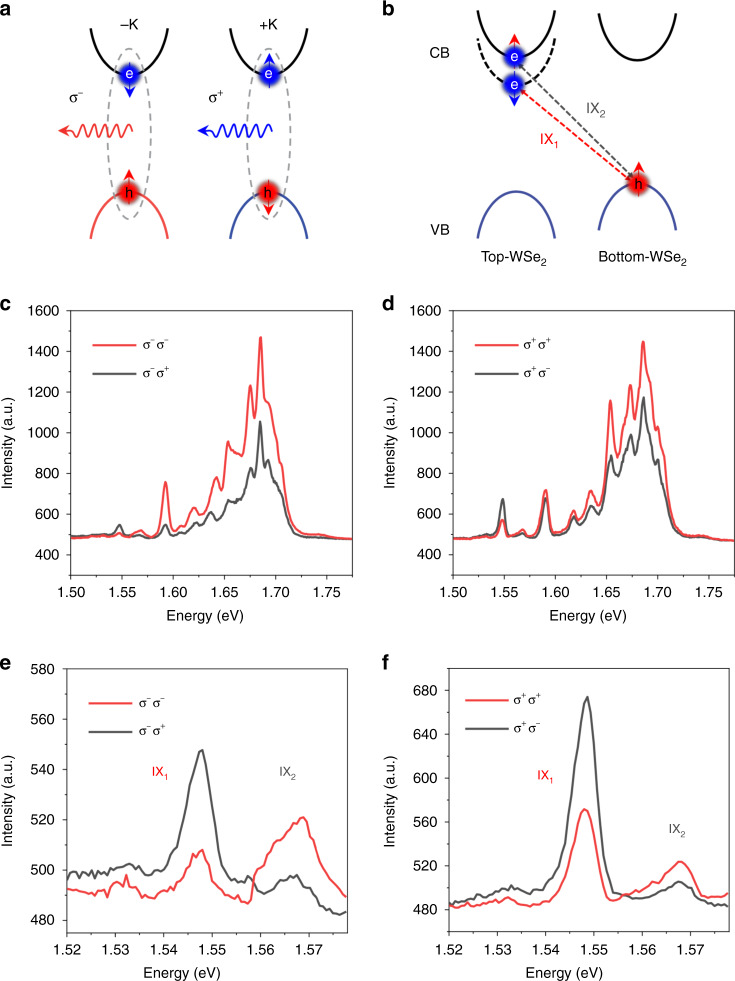
Intralayer and interlayer excitons valley polarization in WSe_2_/WSe_2_ twisted homobilayer. **a** Illustration of the valley optical selection rule, where the blue (red) sphere indicates electrons (holes) in the conduction (valence) band. The arrow represents spin up or down. The red (blue) wavy arrow indicates the emitted σ^−^ (σ^+^) light. **b** illustration of interlayer excitons transition in the twisted WSe_2_/WSe_2_ homobilayer. The red and gray dashed double arrows indicate the radiative recombination of the interlayer excitons IX_1_ and IX_2_. **c** and **d** represent the circularly polarized PL spectra in the T-HB region under σ^−^ and σ^+^ excitation, respectively. **e** and **f** are the circularly polarized PL spectra of interlayer excitons under σ^−^ and σ^+^ excitation, respectively. The interlayer excitons appear opposite circular polarization. σ^*x*^σ^*y*^ shows excitation with σ^*x*^ pump and detection with σ^*y*^ circular polarization. The measured temperature in (**c**–**f**) is 8 K

To better understand the circular polarization properties of moiré intralayer excitons, we studied the polarization-resolved PL spectrum of T-HB at 8 K. According to the optical selection rule shown in Fig. 5a, the valley is coupled to the PL circularly. Hence, the characteristic of the valley can be characterized by the circular polarization-resolved PL measurement. When excited with σ^−^ (or σ^+^) pump light, the co- (σ^−^ detection) and cross- (σ^+^ detection) circularly polarized PL spectra were collected^30^. Here we define circular polarization as^31^

2 $$P_{\sigma - } = [I(\sigma ^ - )] - I(\sigma ^ + )/[I(\sigma ^ - ) + I(\sigma ^ + )]$$

where *I*(σ^±^) represents the measured PL intensity. Figure 5b shows the IX transitions of the type-II structure. Due to the spin-orbit coupling effect^32^, the conduction band of WSe_2_ splits into spin-up and spin-down energy levels, where electrons in these two energy levels combine with holes in the adjacent layer valence band to form interlayer excitons IX_1_ and IX_2_. Figure 5c shows that when excited with σ^−^ pump light, the emission intensity detected with σ^−^ is stronger than that detected with σ^+^ when the energy is in the range of 1.55–1.73 eV. Similarly, Fig. 5d presents that the emission intensity detected with σ^+^ is stronger than that detected with σ^−^ when excited with σ^+^ pump light. The difference in emission intensity detected by σ^+^ and σ^−^ is caused by the valley optical selection rule^33–35^. Under the excitation of σ^−^ pump light, carriers are generated in the −K valley and recombined to emit σ^−^ light, while no carriers are generated in the +K valley. However, the emission light is detected by σ^+^ due to intervalley scattering. Meanwhile, only carriers in the +K valley are excited when excited by σ^+^ pump light.

Figure 5e, f shows that the PL spectra of the T-HB region at 8 K appear with two distinct emission peaks around 1.55 eV, consistent with the energy range of the interlayer exciton peaks^36–40^. Meanwhile, the energy difference between the two IX peaks is 20 meV, which is also in good agreement with previous reports^38,41^. The IX peak splitting phenomenon is attributed to the spin-orbit coupling effect of the conduction band of WSe_2_^32,38^. The WSe_2_/WSe_2_ T-HB forms a type-II band alignment, and Fig. 5b shows the IX transition of the type-II structure. Because of the spin-orbit coupling effect, the conduction band of WSe_2_ is split into spin-up and spin-down energy levels, and electrons in these two energy levels combine with holes in the valence band of adjacent layers to form interlayer excitations IX_1_ and IX_2_ due to forceful Coulomb interactions^38,40^. The lifetime of these IXs is much longer than that of direct excitons, which is critical for long-lived exciton devices^42–44^. Interestingly, under the excitation of circularly polarized light, it can be clearly observed that both IX_1_ and IX_2_ exhibit cross-polarization^31^. The results indicate that the spin directions of the conduction band and valence band energy levels of the interlayer excitons IX_1_ are opposite, while the spin directions of the energy levels of the interlayer excitons IX_2_ are the same opposite as shown in Fig. 5b.

We have prepared a high-quality WSe_2_/WSe_2_ HB with a small twist angle, and successfully observed moiré intralayer excitons at 8 K by measuring the PL spectrum. The multiple split peaks with energies ranging from 1.55 to 1.73 eV are different from that of the monolayer WSe_2_ exciton peaks, which were caused through the trapping of intralayer excitons by the moiré potential. In addition, two distinct IX peaks appear at about 1.55 eV, with an energy difference of 20 meV, which is caused by the spin-orbit coupling effect. Furthermore, the effects of changes in temperature and laser power, as well as the valley polarization on the moiré excitons were performed and the key observed spectral features were explained. Our results provide new opportunities for quantum emitters and many-body phenomena.

## Materials and methods

### Sample fabrication

Monolayer WSe_2_ and hBN flakes were obtained from bulk crystals (HQ graphene) by mechanical exfoliation. The T-HB samples were prepared under the microscope using the tear-and-stack technique^7,22^. The sample preparation process (including mechanical exfoliation, tearing, and stacking) was performed in a glove box transfer platform under a fully enclosed nitrogen atmosphere with water content below 0.02 ppm and an oxygen content below 0.2 ppm. A glass slide covered with a piece of polydimethylsiloxane (PDMS) and a drop of polycarbonate (PC) was used first to pick up a part of the monolayer WSe_2_, followed by twisting the remaining part of the monolayer WSe_2_ at a small angle, and then stack the picked-up first part of the monolayer WSe_2_ vertically on to the remaining monolayer WSe_2_, and then pick it up together. The next step is to transfer the WSe_2_ bilayer onto the hBN sheet and peel off the PC glue. The PC glue was dissolved in dichloromethane at room temperature for 2 h. Finally, the prepared samples were annealed in an argon atmosphere at 300 °C for 3 h to make the sample interface cleaner. There were no visible cracks, wrinkles, or contamination in the optical image, indicating that we successfully prepared high-quality T-HB samples. It should be mentioned that the sample preparation may confine water vapor at the interface, but the proportion of water vapor in the total area is negligible compared to the area of the T-HB region.

### Optical measurements

All-optical data were measured on the WITec Alpha 300R system, which has 50× objective lenses and a light spot of nearly 1 μm. Before the optical measurement, the pressure in the cryogenic chamber should be kept below 10^−5^ Pa, and then the chamber was heated to 350 K for 20 min to remove the water vapor. Finally, optical measurements were performed on the WITec Alpha 300R Raman system when the sample temperature reached 8 K. The model number of the cryogenic refrigeration system is C04-005-044 from Cryo Industries of America. The excitation light source of the SHG signal was a 1064 nm pulsed laser, and the excitation wavelength of the excitation light source measured by other data was 532 nm (2.33 eV). The angle data shown in Fig. 1e is fitted by a sine function, *y* = *y*_0_ + A*sin (B*α + *φ*), where *y* is the intensity of SHG, *y*_0_, *A*, *B* are constants, *α* is the angle of rotation of the half-wave plate, and *φ* is a fitting parameter defining the relative orientation of TMD crystal lattices. The twist angle between the two monolayers can be deduced by fitting the resulting function.
